# Adaptation constraints in scenarios of socio-economic development

**DOI:** 10.1038/s41598-023-46931-1

**Published:** 2023-11-24

**Authors:** Emily Theokritoff, Nicole van Maanen, Marina Andrijevic, Adelle Thomas, Tabea Lissner, Carl-Friedrich Schleussner

**Affiliations:** 1 Climate Analytics, Berlin, Germany; 2 Geography Department & IRI THESys, Humboldt University of Berlin, Berlin, Germany; 3 International Institute for Applied Systems Analysis, Laxenburg, Austria; 4 University of The Bahamas, Nassau, Bahamas

**Keywords:** Projection and prediction, Climate-change adaptation

## Abstract

Climate change adaptation is paramount, but increasing evidence suggests that adaptation action is subject to a range of constraints. For a realistic assessment of future adaptation prospects, it is crucial to understand the timescales needed to overcome these constraints. Here, we combine data on documented adaptation from the Global Adaptation Mapping Initiative with national macro indicators and assess future changes in adaptation constraints alongside the Shared Socioeconomic Pathways, spanning a wide range of future socio-economic development scenarios. We find that even in the most optimistic scenario, it will take until well after 2050 to overcome key constraints, which will limit adaptation for decades to come particularly in vulnerable countries. The persistence of adaptation constraints calls for stringent mitigation, improved adaptation along with dedicated finance and increasing efforts to address loss and damage. Our approach allows to ground truth indicators that can be further used in climate modelling efforts, improving the representation of adaptation and its risk reduction potential.

## Introduction

The gap between adaptation implementation and levels needed to adjust to the effects of climate change is growing^1,2^. This gap can partly be explained by adaptation constraints, which the Intergovernmental Panel on Climate Change (IPCC) defines as “factors that make it harder to plan and implement adaptation actions”^3^. There is clear evidence of adaptation constraints hindering adaptation progress globally and at all spatial scales—from individual households to national governments to regional institutions^3,4^. Nevertheless, measuring adaptation—including constraints—remains a challenge in the scientific community and beyond, as outlined in the recent Sixth Assessment Report of the IPCC^3^.

A better understanding of factors enabling or constraining adaptation is important for improved adaptation but also crucial for risk assessments under scenarios of future climate change. Future climate impacts depend not only on changing climate hazards but fundamentally also on the level of adaptation. Indeed, a large number of sectoral impact studies imply that assumptions about levels of adaptation and changes in socio-economic conditions are as important for the level of future risks as different levels of future warming^3,5^. Approaches integrating adaptation constraints in scenarios of sectoral adaptation illustrate the strong dependence on socio-economic development^6,7^. Yet assumptions about adaptation in future risk assessments often remain very stylised and ‘ad-hoc’, assuming optimal states of adaptation or no adaptation at all, resulting in an under- or over-estimation of the risk reduction potential of adaptation^8^. A key challenge to an improved integration of adaptation and its constraints in the context of global modelling approaches is that available global datasets are often top-down in nature and notoriously hard to validate at national, regional and local levels^9^. This is partly due to the lack of cross-scale approaches combining multiple sources of information^9^. As a result, adaptation is often not adequately taken into account in climate modelling efforts^6,7^.

To date, constraints have mostly been studied at the local and project level, however, national adaptation processes and policies also experience a variety of constraints which significantly hinder the effectiveness and efficiency of adaptation^10^. Case study research is invaluable in the depth of information that it provides for a specific context, but there is a simultaneous need for comparative and global analyses to account for adaptation progress^11^. In the past, path dependencies in the field of adaptation have shown that for example institutions that are resistant to change can be drivers of further constraints and limits to adaptation^12^. Here, we use a novel source of bottom-up evidence, namely a big database of scientific literature on adaptation, to validate top-down socio-economic macro-indicators in the context of constraints to climate adaptation. This allows us to assess how adaptation constraints may develop in the future under different scenarios of socio-economic development and at what timescales such constraints might be overcome.

Bottom-up empirical evidence on adaptation progress is provided by the Global Adaptation Mapping Initiative (GAMI) (https://globaladaptation.github.io). Combining machine learning and systematic literature review techniques, GAMI screened more than 48,000 articles, resulting in a unique database of 1,682 qualitatively coded scientific papers on the current state of implemented adaptation^13^. For each coded article on adaptation, specific information on whether constraints to adaptation were mentioned or not was recorded, reflecting whether constraints were a topic encountered during the study and allowing to understand the current landscape of adaptation constraints. Across the GAMI database, the most reported constraints are governance/institutions/policy ones (mentioned in 54% of the articles which identify constraints), followed by finance constraints (49%), which arise alongside human capacity, information and social/cultural constraints^4^. Finance and governance constraints are found to be key challenges for governments (from national to local levels) and civil society (mainly at the sub-national and local levels)^4^.

The top-down socio-economic indicators are based on quantified dimensions of the Shared Socioeconomic Pathways (SSPs)^14^. The SSPs are a set of five broad narrative-based scenarios of future development over the twenty-first century^14^, of socio-economic dimensions such as population^15^, Gross Domestic Product (GDP)^16,17^, education^15^, and from recent extensions, governance^18^ and gender equality^19^. These dimensions are related to key adaptation constraints, such as financial and economic resources^20^, insufficient quality of governance and institutions^18^, gender inequality^19^ and lack of education^21^. Indeed, lack of effective governance or presence of corruption is related to governance/institutions/policy constraints as it hinders adaptation from the planning to the implementation phase due to low prioritization, diversion of resources away from adaptation needs, red-tape and many more aspects that manifest on different levels of governance^18^. Gender inequality relates to finance constraints as uneven access to resources, cultural norms and entrenched social structures can hinder adaptation, on the individual as well as the broader societal level^19^*.* Education infrastructure (both social and material) is related to governance/institutions/policy constraints, as education can reduce vulnerabilities and research-based adaptation learning support can trigger social and policy change^22^. For example, education has been found to reduce disaster-related mortality as it lowers vulnerability before, during and after the disaster^21^.

When exploring potential futures, we decide to focus on three of the five SSP scenarios, namely SSP1, SSP2 and SSP3, which capture a comprehensive range of alternative pathways, relevant to explore in the context of constraints. SPP1, the ‘sustainability’ scenario, is characterised by low challenges in adaptation and mitigation and emphasises on rapid and inclusive development respecting environmental boundaries. SSP2, the ‘middle of the road’ scenario, maintains current challenges to adaptation and mitigation and illustrates a world in which social, economic and technological trends do not change markedly from historical patterns. SSP3, the ‘regional rivalry’ scenario, also known as ‘rocky road’, is characterised by high challenges for both mitigation and adaptation and anticipates a fragmented world where resurgent nationalism dominates and socioeconomic development stalls^14^. SSP1 and SSP5, and SSP3 and SSP4 are pair-wise similar with regards to their assumptions about future socio-economic development and challenges to adaptation, which is why we exclude both SSP5 and SSP4 from our analysis for the sake of improved readability.

In this study, we establish bottom-up informed national constraint level proxies, largely based on aggregated local case study literature from GAMI. Secondly, we ground truth existing national level socio-economic indicators with these proxies. Lastly, we explore the temporal evolution of theses proxies and socio-economic indicators in the twenty-first century alongside three SSPs. This provides insights into the current presence of constraints on a global level, how they relate to macro socio-economic indicators and the timescales needed to improve the socio-economic dimensions crucial for adaptation.

## Results

### GAMI national adaptation constraint proxies

**Figure 1:**
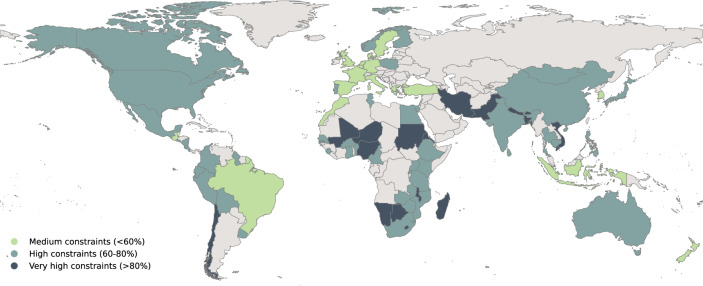
World map of adaptation constraint proxies. Country level assignment to medium, high and very high constraints categories are indicated by different colours. Only countries with 5 or more papers in the database are included. See supplementary Fig. 2 for the absolute numbers of papers on constraints in the GAMI database.

To measure how adaptation constraints may evolve and be overcome, a present-day baseline needs to be established. Despite the highly context-specific nature of adaptation constraints, the comprehensiveness of the GAMI database allows us to create national constraint level proxies. We calculate the percentage of adaptation papers identifying constraints for 83 countries and define three broad categories: (1) medium evidence of constraints when less than 60% of assessed literature on that country identifies constraints (17 countries); (2) high evidence of constraints when between 60 and 80% of assessed literature identifies constraints (47 countries) and (3) very high evidence of constraints when above 80% of assessed literature identifies constraints (19 countries, compare also Fig. 1). Broadly, countries with low socio-economic development tend to fall in the highly constrained categories whereas the countries with higher socio-economic development are often less constrained. Overall, the results align with other existing efforts of quantifying adaptation constraints^23^.

### Comparisons between national adaptation constraint levels and socio-economic indicators

**Figure 2:**
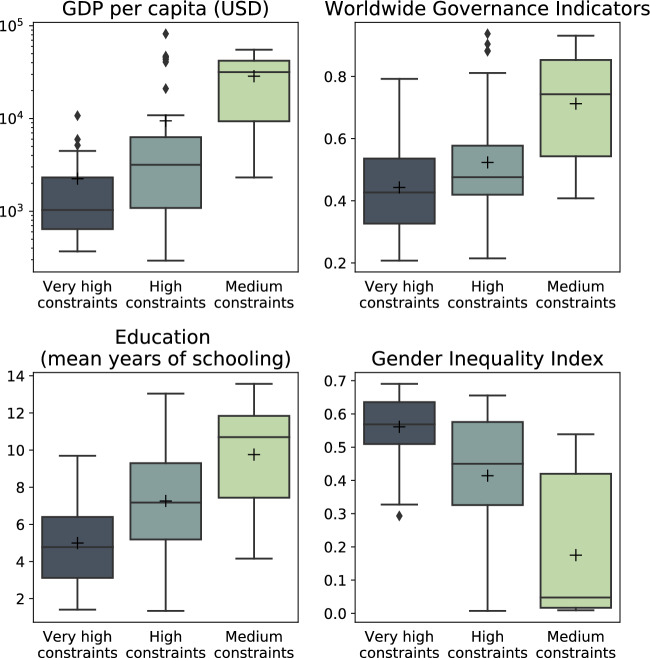
Socio-economic indicators per constraint level. Box plots showing GDP per capita, Worldwide Governance Indicators, Education (mean years of schooling), Gender Inequality Index) per constraint level category. The Worldwide Governance Indicators range from 0 to 1, with higher values indicating better governance. The Gender Inequality Index also ranges from 0 to 1, with higher values indicating great gender inequality.

Constraints to adaptation are closely intertwined with socio-economic factors^3^. To put the present-day adaptation constraint proxies into perspective, we relate them to quantified dimensions of the SSPs for which projections until 2100 are available (Fig. 2). We first show the relationship between constraint levels and GDP per capita. Countries with higher levels of GDP per capita report lower levels of constraints, confirming that poorer countries face more difficulties to adapt to climate change (Fig. 2). On average, countries in the medium constraints category also show better governance and education and lower gender inequality (Fig. 2). These four socio-economic indicators broadly cover six out of the eight constraint types defined by the IPCC, namely economic, social/cultural, human capacity, governance/institutions/policy, financial and information/awareness/technology (but not physical and biological) which are also represented in the GAMI database^4^.

### SSP projections of socio-economic dimensions linked to adaptation constraints

**Figure 3:**
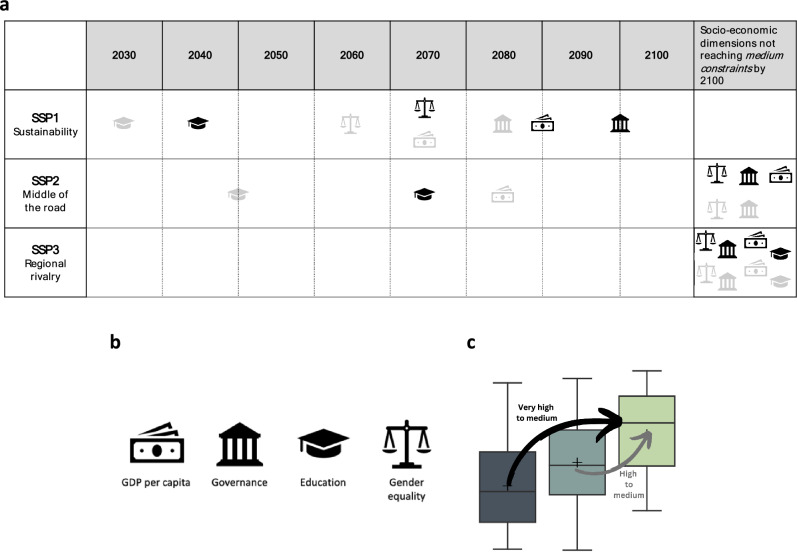
Timescales of socio-economic dimensions reaching medium constraint levels for SSP1, SSP2 and SSP3. (**a**) Timeline of socio-economic dimensions transitioning to medium constraint levels throughout the twenty-first century. The black icons show when very high constraints become medium constraints, the grey icons show when high constraints become medium constraints (**b**) Representation of socio-economic dimension icons. (**c**) Illustration of dimensions moving from very high/high to the medium constraint category (for more information see supplementary Figs. 4 and 5).

Mapping constraints against socio-economic indicators allows us to illustrate SSP-aligned timescales of how adaptation constraints could evolve in the future, moving from very high to high and medium levels of constraints^24^. Specifically, we identify the time periods of category transitions for the constraint dimensions modelled in SSPs (Fig. 3a). Figure 3 shows if and when the four different dimensions would reach medium constraint levels under SSP1, SSP2 and SSP3 by 2100, namely when the very high and high constraint categories for each dimension would transition to the medium constraints category (Fig. 3c).

Using this illustrative approach, we find that it is only under the SSP1 scenario that medium constraints will be achieved across all dimensions in the twenty-first century (Fig. 3a). Transitions happen at very different time periods for the different socio-economic dimensions, with education being the first one to reach medium constraint levels (in 2040) while governance is only projected to reach medium levels towards 2095 (Fig. 3a). This indicates that even under the most optimistic scenarios of socio-economic development, very high constraints to adaptation will prevail well into the second half of the twenty-first century for many countries, especially those in the Global South. In contrast, under SSP3, no constraint transition is achieved for all four dimensions indicating little to no improvements in adaptation for currently very highly and highly constrained countries (Fig. 3a). SSP2 shows that governance and gender equality will not reach medium levels of constraints by 2100 along with GDP for very highly constrained countries (Fig. 3a). Across SSP scenarios, education is the socio-economic dimension that may improve the fastest. According to further tests (Supplementary Figs. 4 and 5), the results are generally robust with regard to the setting of the category thresholds and the qualitative assumptions of our findings.

**Figure 4:**
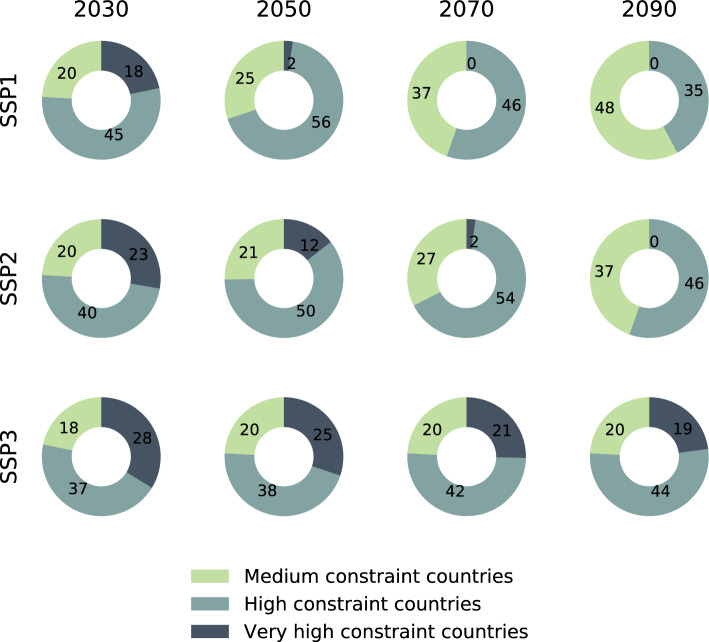
Timescales of countries’ constraint levels. Pie charts showing the number of countries in the medium, high and very high constraint categories in 2030, 2050, 2070 and 2090 and for SSP1, SSP2 and SSP3.

Furthermore, we can then identify illustrative points in time when individual countries may transition from one category to another based on our socio-economic dimensions by SSP scenario. Figure 4 shows in which constraint level category countries would find themselves under SSP1, SSP2 and SSP3 in 2030, 2050, 2070 and 2090. Under SSP1, we find that while countries with very high constraint levels transition by mid-century, not all countries in our sample will reach medium constraint levels by 2090 (42% remain in the high constraint category) (Fig. 4). In contrast, under the SSP3, there are few countries that transition over the twenty-first century (Fig. 4). Under the middle-of-the-road SSP2 scenario, all very high constraint countries will eventually transition in the second half of the twenty-first century, but by 2090, still 55% of the countries are still in the high constraint category (Fig. 4).

## Discussion

Detailed knowledge on socio-economic factors constraining adaptation action is a key building block to understand how national adaptation could be accelerated^4^. To this end, comparing and synthesising bottom-up case studies and top-down approaches provides a promising avenue for more comprehensive assessments^25^. Based on systematically synthesised scientific literature including case studies (from GAMI), we present generalised national level proxies of the current levels of adaptation constraints. Combining these proxies with the socio-economic indicators of the SSPs, we can illustrate future scenario-dependent timescales for overcoming such constraints.

The IPCC AR6 Working Group 2 report finds that “soft limits to some human adaptation have been reached, but can be overcome by addressing a range of constraints, primarily financial, governance, institutions and policy constraints (*high confidence*)”^1^. In this study, we include socio-economic indicators that are closely related to these constraint types (such as GDP per capita and the Worldwide Governance Indicators) and take into account that various constraint types need to be addressed in parallel to improve adaptation^4,25^. We find that baseline values of constraint levels and socio-economic factors are closely related: currently, the higher the constraint levels, the lower the GDP per capita, governance, education and gender equality. With this, we confirm that the ability to adapt is closely intertwined with socio-economic development^26^ and can validate ranges of adaptation constraints based on the bottom-up studies assessed in the GAMI database for 83 countries.

Our results show that key constraints to adaptation are closely interlinked with socio-economic factors which are expected to take a long time to improve over the course of this century. Socio-economic factors are only expected to improve on timescales that match or even exceed full decarbonisation towards achieving global net zero emissions in Paris-Agreement compatible scenarios^27^, even in the most optimistic case investigated here (SSP1)^18,28^. These timescales stand in stark contrast with the urgency of the climate crisis and the dire need for adaptation to avoid reaching hard limits to adaptation and further losses and damages^1^. This challenges a narrative that humanity will adapt to the impacts of climate change that are not reduced by mitigation. If adaptation remains, as our results suggest, substantially constrained for decades to come, a holistic approach is needed going forward with more stringent mitigation efforts reducing the risks and impacts of climate change^26^, improved adaptation and losses and damages being addressed. Additionally, some future climate change impacts, such as sea-level rise, are already locked-in and will persist in the next decades and well beyond the twenty-first century, even if additional greenhouse gas emissions are halted today^29^, further underlining the need for stringent mitigation. This does not mean that adaptation should be de-emphasised. Conversely, substantial scaling-up of adaptation action and in particular financial support for developing countries is urgently required as a gap in adaptation efforts to today’s climate is already more than evident^2^. Our results highlight that the road to adapt to climate change and its impacts might be rockier and much longer than sometimes assumed. Constraints to adaptation are not something temporary that will disappear in the near-term (before 2030) but will persist for decades to come. Nevertheless, our results also highlight the potential for near-term improvements in education, as soon as 2030, as opposed to governance which takes much longer to improve^18^.

Not overcoming constraints increases the likelihood of reaching limits to adaptation, the points at which adaptation actions can no longer avoid intolerable risks^3^. In addition, effectiveness of adaptation measures, in particular widely available ecosystem-based adaptation, may decrease as climate impacts intensify. This study focuses on constraints closely linked to socio-economic dimensions, disregarding biological and physical constraints which would further constrain and limit adaptation. This implies that there is a substantial risk that the emergence of climate impacts can ‘outpace’ socio-economic improvements^26^ to overcome adaptation constraints and that thereby more adaptation limits could be reached^3^. With this mounting pressure, adaptation that is currently predominantly incremental, slow and siloed needs to become increasingly transformational^3^, by moving beyond business as usual and enabling changes to the fundamental attributes of socio-ecological systems^13^. Transformational adaptation also refers to the degree to which adaptation occurs rapidly, reflects major shifts, is implemented widely and challenges limits to adaptation^3,13,30^. This is further emphasised when looking at the results for less optimistic scenarios of socio-economic development. Under the ‘rocky road scenario’ (SSP3), adaptation constraints will persist throughout the twenty-first century, limiting adaptation action in particular in most vulnerable countries. Even under a SSP2 scenario, medium adaptation constraints are not reached by the end of the twenty-first century for most countries.

Using scientific papers as a proxy for the presence of adaptation constraints and some limitations of the GAMI database introduce certain biases. The vast majority of implemented adaptation projects are not represented in the scientific literature, thus limiting the overall coverage of the database. In addition, an identification of a constraint in a study does not tell us how pertinent or extensive this constraint is. The adaptation literature exhibits significant topic biases by geographic location: authors from the Global North often study disasters and development-related topics in countries of the Global South and governance topics often dominate in studies focussed countries of the Global North for example^31^. In addition, there is a scarcity of scientific literature in some highly vulnerable countries^31^ such as Small Island Developing States (SIDS) (see Supplementary Fig. 2). The adaptation constraint proxies could have benefitted from a more complete dataset globally. The scope of our analysis therefore does not allow for assessments of individual countries, but rather illustrates how different socio-economic scenarios of development would continue to pose challenges to adaptation on a global level. In addition, the projections of timescales to overcome key dimensions of adaptation constraints represent extensions of the SSP narratives and are thus not predictions of future development. How the world will actually develop is a question of present and future policy decisions, which may still offer solutions for overcoming such constraints more rapidly than the projections indicate. The sensitivity test shows that the results are somewhat sensitive to the thresholds of the constraint categories, but the qualitative storyline remains unchanged (see Supplementary Figs. 4 and 5).

The choice of analysing adaptation constraints at the national level, through the use of national level proxies and indicators, does not provide a granular picture of adaptation constraints occurring on more local levels and certainly does not illustrate how constraints can be overcome in practice. Indeed, in a country with low levels of constraints to adaptation at the national level, it is not excluded that specific adaptation projects on the local level face very high constraints due to context specific challenges. Accounting for these sub-national divergences would nevertheless only render our results conservative. Despite these limitations, we report good agreement of the bottom-up evidence for adaptation constraints and top-down indicators on the level of country groupings (compare Fig. 2), which gives us confidence in the robustness of our key findings. Future research could focus on the linkages between socio-economic development and constraints to adaptation at the sub-national level and explore in-country disparities.

The novel approach deployed in this study is a first step in linking the predominantly local and case study-based adaptation literature with macro indicators used in modelling efforts. Adaptation is currently absent in most quantitative assessments of climate change, resulting in an imperfect picture of the overall challenge that climate change poses and a limited understanding of vulnerabilities across countries and regions^6^. One potential avenue to do this is to embed socio-economic dimensions of adaptative capacity into the SSPs^7^. Various socio-economic indicators can be used to assess adaptive capacity for different sectors and geographies, allowing to better evaluate the risk reduction potential of adaptation^7^. Ground truthing socio-economic indicators that can then further be used in modelling efforts is an important step for the field of adaptation that is in dire need of more quantitative and generalisable metrics to track its progress over time.

## Methods

### Methods protocols

We use the database from the Global Adaptation Mapping Initiative (GAMI), a systematic assessment of academic literature on human adaptation-related responses to climate change (https://globaladaptation.github.io). Detailed protocols describing the methodology used have been published via the Nature Protocol Exchange, and include: Part 1—Introduction and overview of methods (https://doi.org/10.21203/rs.3.pex-1240/v1)^32^, Part 2—Screening protocol (https://doi.org/10.21203/rs.3.pex-1241/v1)^33^ and Part 3—Coding protocol (https://doi.org/10.21203/rs.3.pex-1242/v1)^34^ and in the Supplementary Material of the overarching article^13^.

### GAMI data

GAMI investigates how humans are adapting to climate change on a global scale and analyses the scope, nature and progress of adaptation. More than 48,000 peer-reviewed scientific articles published between 2013 and 2019 (namely between the IPCC’s 5th Assessment Report and the cut-off date of the 6th Assessment Report) were retrieved using search strings for Web of Science, Scops and MEDLINE, revolving around climate change and adaptation. These articles (title and abstract only) were then subsequently screened using machine learning techniques, resulting in a list of 2,032 articles which matched the inclusion criteria. This set of articles was then manually coded by a global network of 126 researchers. 70 questions per article were coded, structured into the seven following thematic sections: (1) general information; (2) who is responding; (3) what responses are documented; (4) what is the extent of adaptation-related responses; (5) are responses reducing risks; (6) adaptation limits (including constraints); and (7) assessing confidence in evidence. Articles were at minimum double-coded and reconciliation of differing codes was undertaken in the R statistical environment^35^. As part of another paper^4^, questions under Section (6) went through an additional round of review and 123 papers were identified as miscoded. The latest round of review was used for this study.

The final GAMI database consists of 1682 papers on adaptation (after a second round of manual screening performed by humans). Out of those, 1239 articles identified constraints, limits and synonyms (data from Section  6). The geographic information of the countries included in the articles was also coded. Only countries with 5 or more papers were included. Based on this, we calculate the country-specific constraint level proxies using the following formula:

$$National\, constraint \,proxy=\frac{Number \,of \,papers \,on\, constraints\, per \,country}{Number\, of \,papers \,on \,adaptation\, per \,country}\times 100$$

After calculating this proxy, we define three categories of constraint levels based on the normal distribution of the data (peaking around 75%) (see Supplementary Fig. 1):

**Table:** 

Constraint level	Threshold values
Medium constraints	< 60% of assessed literature in the country identifies constraints
High constraints	60–80% of assessed literature in the country identifies constraints
Very high constraints	> 80% of assessed literature in the country identifies constraints

Establishing globally applicable categories of what constitutes ‘medium’, ‘high’ and ‘very high’ constraints is to some extent arbitrary. The categorisation starts with ‘medium’ as there are only two countries below 40% of literature identifying constraints (see Supplementary Fig. 1), highlighting widespread constraints globally. It must be noted that the GAMI database provides data points for each individual article and does not differentiate findings between countries if the study covers several geographies. Five outliers were excluded from the dataset, namely countries with less than 10 papers on adaptation with low socio-economic development and medium constraints, or those with high socio-economic development and very high constraints.

In addition, a sensitivity test was conducted for a different set of ranges to assess the implications of our choices:

**Table:** 

Constraint level	Threshold values
Very high constraints	> 70% of assessed literature in the country identifies constraints
High constraints	50–70% of assessed literature in the country identifies constraints
Medium constraints	< 50% of assessed literature in the country identifies constraints

The results of this analysis can be found in the Supplementary Figs. 4 and 5.

### Socio-economic indicators

We select four quantified dimensions of the SSPs that have data until 2100 and are relevant for adaptation, namely: Gross Domestic Product (GDP) per capita, governance, gender inequality and education. For each country, we calculate the baseline values for the four socio-economic indicators by taking the average of the values for the period 2003–2013 (ten years prior the start of the publication period of articles included). Furthermore, we calculate the median values of the four socioeconomic indicators for all countries summarised in each of the three constraint categories (See Supplementary Fig. 3).

### Shared socioeconomic pathways (SSPs)

As a final step, we use the SSP-dependent projections of the four socioeconomic dimensions up until 2100, under SSP1, SSP2 and SSP3. All four indicators have been projected quantitatively alongside the SSPs (see: GDP^16,17,36^, governance^18^, gender inequality^19^ and education^15^). These projections are based on the historical socio-economic indicators used above and are sourced from the same underlying database^24^, ensuring consistency and allowing cross-country comparison.

Firstly, we look at the timescales needed for the median value of the countries in the very high and high constraint categories to transition to the medium constraint category for our four socio-economic dimensions (separately) under SSP1, SSP2 and SSP3 (Fig. 3c; Supplementary Fig. 3). In a second step, we analyse in which categories countries would find themselves up until 2100 under SSP1, SSP2 and SSP3. For this, we assume that a country transitions from one category to the other when three out of the four socio-economic dimensions reach the lower level, namely the median value of this category. Given the width of the category distributions and the considerable overlap (compare Fig. 2), this implies that some countries would transition instantaneously. However, this also means that our approach remains on the optimistic side of overcoming key dimensions of adaptation constraints. Out of the 51 countries in the very high constraint category of the observed baseline, 16 transition instantaneously into the high constraint category when using the modelled baseline values (see Supplementary Fig. 6).

### Supplementary Information

Supplementary Figures.

## Data Availability

The GAMI database is currently not publicly available. Historical GDP per capita was obtained from the Penn World Tables 10.0: https://www.rug.nl/ggdc/productivity/pwt/. The Worldwide Governance Indicators were retrieved here: https://info.worldbank.org/governance/wgi/. Data on mean years of schooling was available through the Data Explorer of the Wittgenstein Centre for Demography and Global Human Capital: http://dataexplorer.wittgensteincentre.org/wcde-v2/. Original data from the Gender Inequality Index (GII) is available through the UNDP website: https://hdr.undp.org/data-center/thematic-composite-indices/gender-inequality-index#/indicies/GII.

## Code availability

The code underlying the results is available at: https://github.com/nicolenicolen/GAMI_constraints_timescales.
