# Supplementary material for: Adaptation constraints in scenarios of socio-economic development
Source: Sci Rep. 2023 Nov 24;13:19604. doi: 10.1038/s41598-023-46931-1 (PMC10673845; doi:10.1038/s41598-023-46931-1)
Supplement: Supplementary file 1 — Supplementary Figures. [file 41598_2023_46931_MOESM1_ESM.docx]

**Supplementary information**

**Supplementary Fig. 1** | **Distribution of constraints.** Share of literature identifying constraints per country.

**Supplementary Fig. 2** | **World map of bottom-up assessed adaptation constraints.** Evidence on the number of papers mentioning constraints in the GAMI database.

**Supplementary Fig. 3** | Baseline median values of socio-economic indicators per constraint level category for the period 2003-2013.

|  | **GDP per capita** | **Governance** | **Education** | **Gender inequality** |
| --- | --- | --- | --- | --- |
| **Very high constraints** | 1032,99 | 0,43 | 4,77 | 0,57 |
| **High constraints** | 3171,62 | 0,48 | 7,17 | 0,45 |
| **Medium constraints** | 31699,67 | 0,74 | 10,70 | 0,05 |

**Supplementary Fig. 4** | Sensitivity test of the baseline median values of socio-economic indicators per constraint level category for the period 2003-2013.

| **Categories** | **New ranges** | **Number of countries** |
| --- | --- | --- |
| Very high constraints | >70% of assessed literature in the country identifies constraints | 47 |
| High constraints | 50-70% of assessed literature in the country identifies constraints | 27 |
| Medium constraints | <50% of assessed literature in the country identifies constraints | 9 |

|  | **GDP per capita (% change with original value)** | **Governance**  **(% change with original value)** | **Education**  **(% change with original value)** | **Gender inequality**  **(% change with original value)** |
| --- | --- | --- | --- | --- |
| **Very high constraints** | 1347,48 (30%) | 0,45 (5%) | 5,83 (22%) | 0,57 (0%) |
| **High constraints** | 6749,03 (113%) | 0,53 (10%) | 7,87 (10%) | 0,38 (-15%) |
| **Medium constraints** | 40238,86 (27%) | 0,85 (14%) | 11,85 (11%) | 0,02 (-60%) |

**Supplementary Fig. 5** | **Socio-economic timescales.** Table providing information on the year during which the socio-economic dimensions will move from the very high/high constraint level to the medium one. The values in parentheses correspond to the results of the sensitivity test (see thresholds in Supplementary Fig. 4).

| **Timescales from the very high to the medium constraint level** | | | |
| --- | --- | --- | --- |
| **Socio-economic dimensions** | **SSPs** | **Year when medium constraint level reached** | **Highest value reached by 2100** |
| GDP per capita | SSP1 | 2085  (Beyond 2100) | (39876,30) |
|  | SSP2 | Beyond 2100 (Beyond 2100) | 31273,28  (29110,49) |
|  | SSP3 | Beyond 2100 (Beyond 2100) | 15867,27  (13115,90) |
| Governance | SSP1 | 2095  (Beyond 2100) | (0,74) |
|  | SSP2 | Beyond 2100 (Beyond 2100) | 0,67  (0,66) |
|  | SSP3 | Beyond 2100 (Beyond 2100) | 0,54  (0,53) |
| Education | SSP1 | 2040  (2050) | - |
|  | SSP2 | 2070  (2085) | - |
|  | SSP3 | Beyond 2100 (Beyond 2100) | 8,35  (9,01) |
| Gender inequality | SSP1 | 2070  (2080) | - |
|  | SSP2 | Beyond 2100 (Beyond 2100) | 0,11  (0,09) |
|  | SSP3 | Beyond 2100 (Beyond 2100) | 0,46  (0,43) |

| **Timescales from the high to the medium constraint level** | | | |
| --- | --- | --- | --- |
| **Socio-economic dimensions** | **SSPs** | **Year when medium constraint level reached** | **Highest value reached by 2100** |
| GDP per capita | SSP1 | 2070  (2055) | - |
|  | SSP2 | 2080  (2060) | - |
|  | SSP3 | Beyond 2100  (2095) | 24025,60 |
| Governance | SSP1 | 2080  (Beyond 2100) | (0,82) |
|  | SSP2 | Beyond 2100 (Beyond 2100) | 0,71  (0,77) |
|  | SSP3 | Beyond 2100 (Beyond 2100) | 0,61  (0,69) |
| Education | SSP1 | 2030  (2040) | - |
|  | SSP2 | 2045  (2060) | - |
|  | SSP3 | Beyond 2100 (Beyond 2100) | 10,24  (11,10) |
| Gender inequality | SSP1 | 2060  (2065) | - |
|  | SSP2 | Beyond 2100 (Beyond 2100) | 0,06  (0,03) |
|  | SSP3 | Beyond 2100 (Beyond 2100) | 0,32  (0,22) |

**Supplementary Fig. 6** | **Observed and modelled baselines.** The observed baseline is calculated based on the values of the socio-economic indicators for 2008 for each country. The modelled baseline is derived from the start of the SSP projections in the year 2015. The latter is used to calculate the timescales of countries’ constraint levels (Fig.4).
